# *EVI1 *activation in blast crisis CML due to juxtaposition to the rare 17q22 partner region as part of a 4-way variant translocation t(9;22)

**DOI:** 10.1186/1471-2407-8-193

**Published:** 2008-07-09

**Authors:** An De Weer, Bruce Poppe, Barbara Cauwelier, Andre Carlier, Jan Dierick, Bruno Verhasselt, Jan Philippé, Nadine Van Roy, Frank Speleman

**Affiliations:** 1 Centre for Medical Genetics Gent (CMGG), Ghent University Hospital, Ghent, Belgium; 2 Department of Hematology, AZ Sint-Jan AV, Brugge, Belgium; 3 Department of Hematology, AZ Maria-Middelares Hospital, Ghent, Belgium; 4 Department of Clinical Biology, AZ Maria-Middelares, Ghent, Belgium; 5 Department of Clinical Chemistry, Microbiology and Immunology, Ghent University Hospital, Ghent, Belgium; 6 Centre for Molecular Diagnostics, Ghent University Hospital, Ghent, Belgium

## Abstract

**Background:**

Variant translocations t(9;22) occur in 5 to 10% of newly diagnosed CMLs and additional genetic changes are present in 60–80% of patients in blast crisis (BC). Here, we report on a CML patient in blast crisis presenting with a four-way variant t(9;22) rearrangement involving the *EVI1 *locus.

**Methods:**

Dual-colour Fluorescence In Situ Hybridisation was performed to unravel the different cytogenetic aberrations. Expression levels of *EVI1 *and *BCR/ABL1 *were investigated using real-time quantitative RT-PCR.

**Results:**

In this paper we identified a patient with a complex 4-way t(3;9;17;22) which, in addition to *BCR/ABL1 *gene fusion, also resulted in *EVI1 *rearrangement and overexpression.

**Conclusion:**

This report illustrates how a variant t(9;22) translocation can specifically target a second oncogene most likely contributing to the more aggressive phenotype of the disease. Molecular analysis of such variants is thus warranted to understand the phenotypic consequences and to open the way for combined molecular therapies in order to tackle the secondary oncogenic effect which is unresponsive to imatinib treatment.

## Background

Chronic myeloid leukemia (CML) has a typical indolent chronic phase that may last several years but will ultimately progress into acute myeloid leukemia (AML) or acute lymphoid leukemia (ALL). The typical associated translocation t(9;22)(q34;q11), leading to the *BCR/ABL1 *fusion gene and constitutive activation of the *ABL1 *tyrosine kinase on 9q34, is considered to be the initial transforming event [1]. Instead of the classical t(9;22) translocation, cryptic and variant translocations occur in about 5 to 10 % of all CML cases. CML in blast crisis is often accompanied by the presence of additional chromosome aberrations [2]. Amongst those, activation of the *EVI1 *gene has been reported in a small percentage of patients [3]. Ectopic expression of the *EVI1 *gene is usually due to recurrent 3q26 translocations such as the t(3;21)(q26;q22) (*AML1/EVI1*) and the inv(3;3)(q21q26). Transcriptional activation of *EVI1 *can also occur in the absence of genomic rearrangement at this locus [4]. In general, *EVI1 *upregulation confers a poor prognosis in hematological malignancies [5].

In this study, we describe how the molecular characterization of the translocation breakpoints of a variant t(9;22) in a patient with CML in blast crisis lead to the discovery of involvement of the *EVI1 *locus. We also discuss the importance of the study of secondary genomic events present or occurring during blast crisis with respect to the development of strategies for treatment of CML patients in blast crisis.

## Methods

### Patient material

A diagnostic bone marrow sample was obtained from a patient who presented with features of chronic myeloid leukemia in blast crisis. The patient died nine months after diagnosis.

This study was approved by the Ethical Committee of the Ghent University Hospital (2003/273).

### Cytogenetic analysis

Cytogenetic analysis of the diagnostic bone marrow sample was performed according to standard methods. A 24-hour bone marrow culture was performed and chromosomes were G-banded with a trypsin-Giemsa stain (GTG-banding). Fifteen metaphases were analyzed and the karyotype was described according to the International System for Human Cytogenetic Nomenclature [6].

### Fluorescence in situ hybridisation analyses

Dual-colour Fluorescence In Situ Hybridisation (FISH) was performed on the diagnostic bone marrow sample using a t(9;22) specific *BCR/ABL1 *dual color, dual fusion probe (Abbott Vysis, Germany). Commercial Whole Chromosome Paints (WCP) for chromosomes 3, 9, 17 and 22 (Metasystems, Germany) were used to unravel the translocations in the bone marrow sample. A commercial centromeric probe for chromosome 3 (Abbott Vysis, Germany) was used to facilitate identification of chromosome 3.

Detection of *EVI1 *rearrangement was done using the following BAC clones: RP11-694D5 (position 169.8 Mb, centromeric of *EVI1*), RP11-82C9 (position 170.3 Mb, *EVI1 *overlapping) and RP11-362K14 (position 171.1 Mb, telomeric of *EVI1*) (NCBI genome browser Build 36.1). All non-commercial clones were acquired from the Sanger Wellcome Trust Institute, Hinxton, Cambridge (United Kingdom). The *EVI1 *specific probes were labelled with biotin-16-dUTP (Roche Diagnostics, Belgium) and digoxigenin-12-dUTP (Roche Diagnostics, Belgium) and FISH analysis was performed as previously described [7,8]. Biotin labelled probes were detected with Fluorescein IsoThioCyanate (FITC)-conjugated anti-biotin antibodies (Invitrogen, Belgium) and digoxigenin labelled probes with TetramethylRhodamine IsoThioCyanate (TRITC)-conjugated anti-digoxigenin antibodies (Roche Diagnostics, Belgium). A minimum of 100 nuclei and a minimum of 5 metaphases were scored using a fluorescence microscope (Axioplan 2, Zeiss, Belgium) and images were captured using a black and white CCD camera and images were processed with the ISIS software program (MetaSystems, Germany).

### Real-time quantitative RT-PCR

Total RNA was extracted from the diagnostic bone marrow sample using the Trizol LS reagent (Invitrogen, Belgium) according to the manufacturer's recommendations. cDNA was prepared from 2 μg of total RNA with the iScript cDNA Synthesis Kit (Bio-Rad, Belgium) according to the manufacturer's instructions.

Real-time quantitative RT-PCR (qRT-PCR) for the *EVI1 *(forward: 5'-GTACTTGAGCCAGCTTCCAACA-3', reverse: 5'-CTTCTTGACTAAAGCCCTTGGA-3', exon 1b-2), c*EVI1 *(forward: 5'-ACCCACTCCTTTCTTTATGGACC-3', reverse: 5'-TGATCAGGCAGTTGGAATTGTG-3', exon 8–9), *MDS1/EVI1 *(forward: 5'-GAAAGACCCCAGTTATGGATGG-3', reverse: 5'-GTACTTGAGCCAGCTTCCAACA-3', exon 2 *MDS1 *– exon 2 *EVI1*) and *MDS1 *(forward: 5'-GAAAGACCCCAGTTATGGATGG-3', reverse: 5'-TCTCTTCCCCAAATACAACCAAG-3', exon 2–3) transcripts was performed as previously described [9,10]. Cell line K562 was used as a positive control and reference, its *EVI1 *expression level was set to 1.

QRT-PCR for the *BCR/ABL1 *fusion transcript was done according to the Europe Against Cancer program (EAC) protocol [11].

## Results

**Figure 1:**
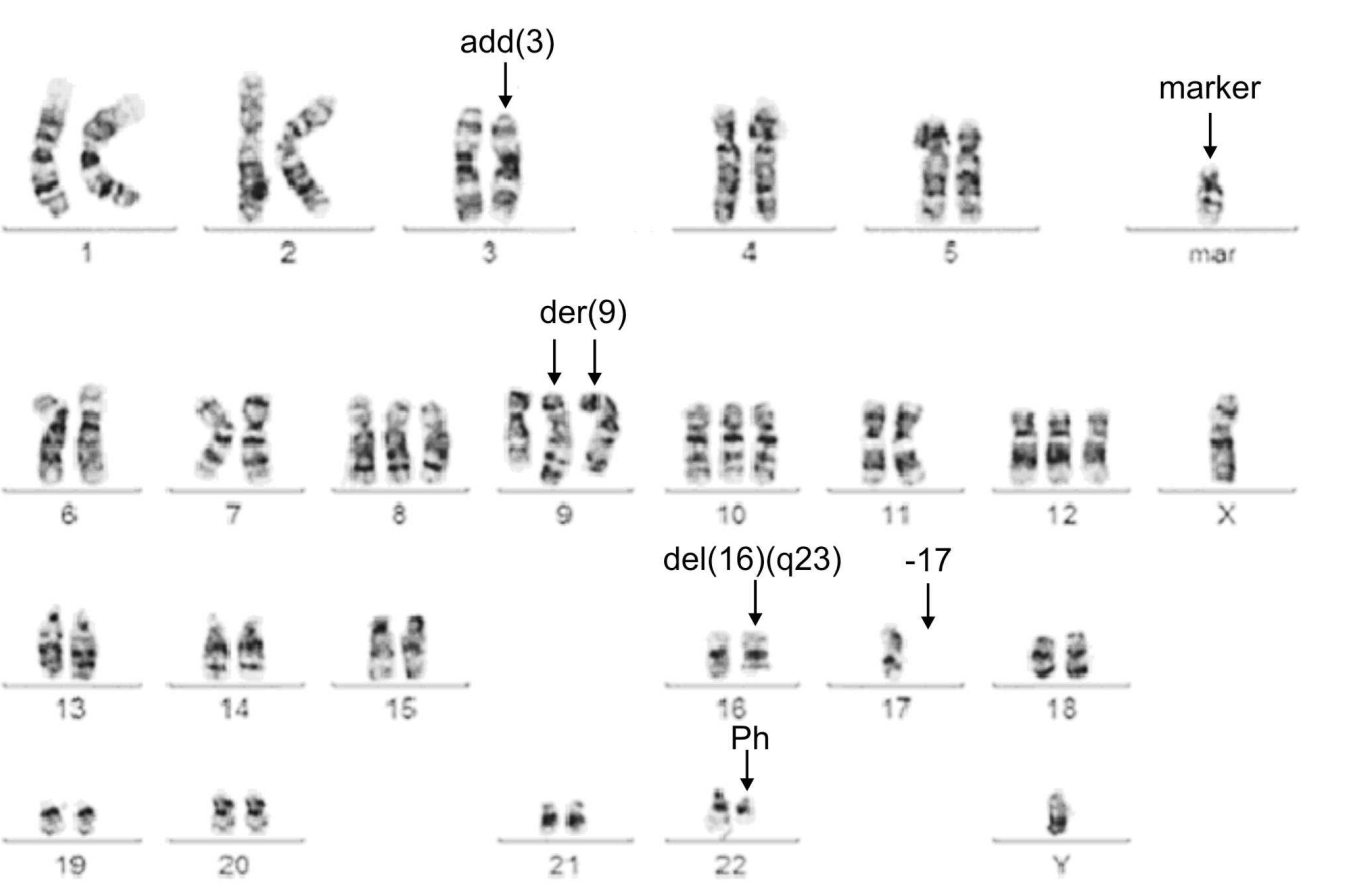
**Representative karyotype from bone marrow of the patient**. Arrows indicate a derivative chromosome 3 with an addition, a marker chromosome, two derivative chromosomes 9 (der(9)), del(16)(q23), the loss of a chromosome 17 and a Ph-chromosome.

Detailed cytogenetic analysis of 15 G-banded metaphases from the diagnostic bone marrow revealed the following karyotype: 50, XY, add(3)(q26),+8, add(9)(q34),+add(9)(q34),+10,+12, del(16)(q23),-17, der(22)t(9;22)(q34;q11),+mar [cp15] (Fig. 1).

**Figure 2:**
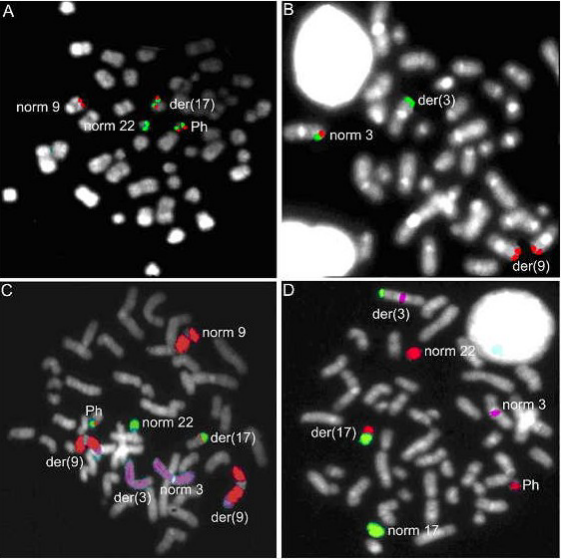
**FISH analysis on metaphases from bone marrow slides**. (A) FISH with the dual color, dual fusion *BCR/ABL1 *probe generates two fusion signals (*ABL1 *red and *BCR *green): one on a marker chromosome [der(17)] and one on the Ph-chromosome, one red signal on a normal chromosome 9 and one green signal on a normal chromosome 22. (B) *EVI1 *specific probes RP11-362K14 (red, distal from *EVI1*) and RP11-82C9 (green, *EVI1 *overlapping) showed a co-localized red-green signal on the normal chromosome 3, a green signal on the derivative chromosome 3 [der(3)] and two red signals translocated onto identical but unidentified chromosomes [der(9)]. (C) FISH with WCPs for chromosomes 3 (purple), 9 (red) and 22 (green), revealed one normal chromosome 9, two derivative chromosomes 9 [der(9)], a der(17) containing a piece of chromosome 22 and chromosome 9, a normal chromosome 3 (norm 3), a derivative chromosome 3 [der(3)] and a Ph-chromosome. (D) WCP of chromosomes 17 (green) and 22 (red), combined with a purple centromeric probe for chromosome 3, indicated that a translocation between the long arm of chromosome 3 and the long arm of chromosome 17 had occurred as well as a translocation between chromosome 17 and 22.

FISH with the *BCR/ABL1 *dual-color dual-fusion probe revealed two fusion signals together with a single green and red signal in interphase nuclei. In metaphases, one fusion was located on the Philadelphia chromosome whereas the second fusion signal was present on the marker chromosome instead of on the der(9)t(9;22)(q34;q11.2) (Fig. 2A). Real-time quantitative RT-PCR (qRT-PCR) confirmed the presence of the *BCR*/*ABL1 *fusion and revealed a major MBCR transcript (data not shown).

Further FISH characterisation of this t(9;22) variant translocation, revealed involvement of chromosomes 3 and 17. FISH analysis with two probe combinations for the *EVI1 *locus (*EVI1 *centromeric and overlapping; *EVI1 *telomeric and overlapping) revealed a breakpoint 5' and telomeric of *EVI1 *and translocation of the telomeric chromosome 3 segment to the der(9) (Fig. 2B). The translocation of the *ABL1/BCR *segment from the der(9) to the der(17) was accompanied by translocation of the distal 17q segment immediately distal to the *EVI1 *locus on the der(3). FISH with whole chromosome paints confirmed these observations (Fig. 2C–D).

Based on these FISH analyses, the karyotype was revised as 50, XY, der(3)(3pter→q26::17q22→qter),+8, der(9)(9pter→q34::3q26→qter),+der(9)(9pter→q34::3q26→qter),+10,+12, del(16)(q23), der(17)(17pter→q22::9q34q34::22q11→qter), der(22)(22pter→q11::9q34→qter) [cp15].

**Figure 3:**
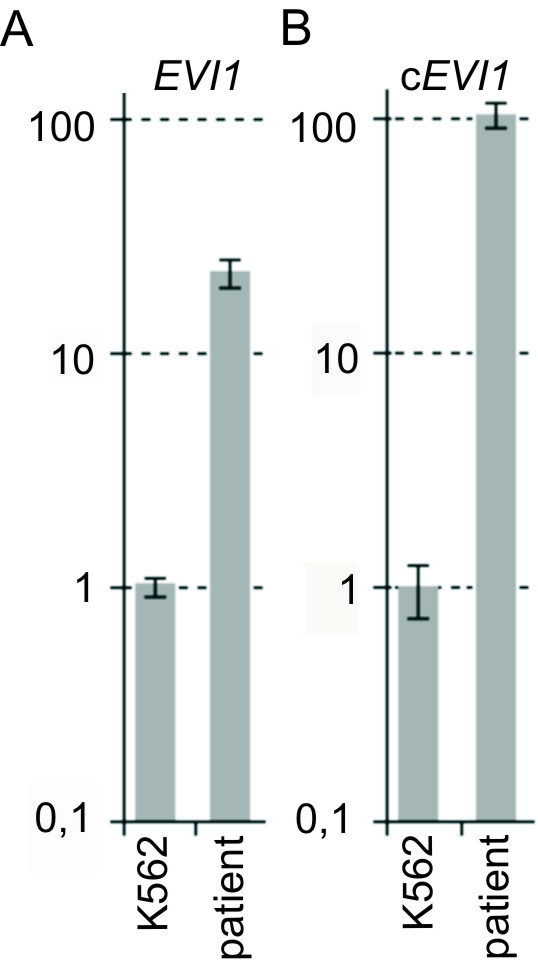
***EVI1 *expression measured by qRT-PCR**. (A) qRT-PCR for *EVI1 *with primers located 5' of the transcript revealed ectopic *EVI1 *expression in the patient after normalization with several household genes, the cell line K562 was used as a positive control, the K562 expression was set to 1, Y-axis in log-scale (B) *EVI1 *qRT-PCR with primers located 3' of the transcript (c*EVI1*) indicated an ectopic expression of a 5' variant *EVI1 *transcript, the cell line K562 was used as a positive control, the K562 *EVI1 *expression was set to 1, Y-axis in log-scale.

Ectopic expression of the full length *EVI1 *transcript was detected with qRT-PCR using the 5' located *EVI1 *primer pair (Fig. 3A). qRT-PCR with the 3' located c*EVI1 *primer pair was able to detect additional ectopic expression 5' *EVI1 *variant transcripts (Fig. 3B). No expression of the *MDS1/EVI1 *or *MDS1 *transcripts could be detected with qRT-PCR (data not shown).

## Discussion

**Figure 4:**
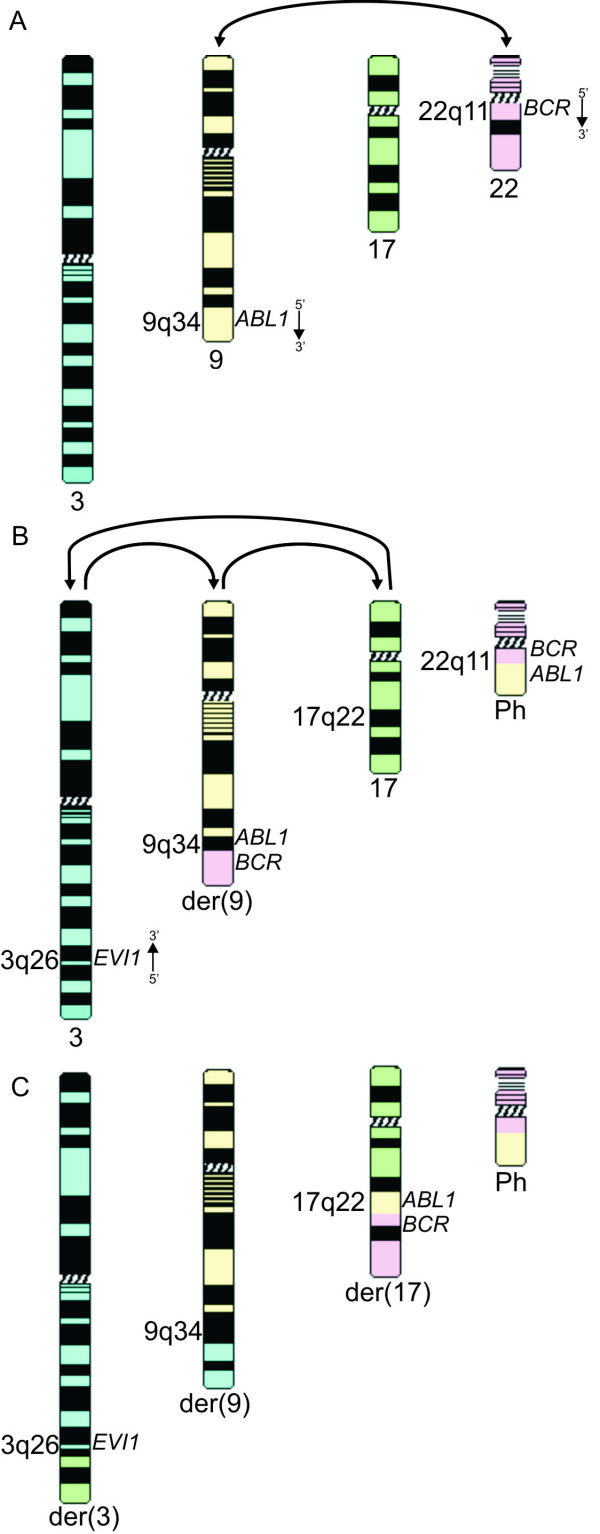
**Schematic representation of possible generation of the 4-way t(3;9;17;22)**. (A) Formation of the t(9;22)(q34, q11). (B) Three-way translocation involving the *EVI1 *locus on 3q26, part of the distal long arm of chromosome 17 and the der(9)t(9;22). (C) Representation of the derivative chromosomes 3, 9, 17 and 22 in our patient.

We describe the molecular characterization of a complex 4-way t(3;9;17;22) translocation, which in addition to *BCR-ABL1 *gene fusion also resulted in *EVI1 *overexpression. Based on FISH and karyotype data we propose a two-step mechanism [12] for generating the complex 4-way translocation, i.e. first the formation of a classical t(9;22), followed by a subsequent three-way translocation involving chromosomes 3, 17 and the derivative chromosome 9. The latter rearrangement was the result of translocation of the *ABL1/BCR *segment from the der(9) to chromosome 17, translocation of the distal 17q segment to the 3q26 locus on chromosome 3 and translocation of the distal part of chromosome 3 to the der(9). Further molecular investigation using FISH indicated that the 3q26 breakpoint located immediately distal of the 5' end of the *EVI1 *locus. As expected qRT-PCR revealed ectopic expression of *EVI1*. A third and final step encompassed the duplication of the der(9) in this patient (Fig. 4A–C).

*EVI1 *overexpression is a well established unfavourable prognostic marker in AML patients, and is often found in progressive or blast crisis CML [4,13,14]. It is therefore likely that, like for the additional alterations such as trisomy 8 and isochromosome 17q, the observed *EVI1 *overexpression contributes to the aggressive phenotype of the blastic CML phase. The presence of the additional cytogenetic abnormality del(16)(q23) in this patient could also negatively influence disease progression.

Apart from the known recurrent secondary changes occurring during progression or blast phase in CML, most additional rearrangement breakpoints have remained unexplored at the molecular level. It can be assumed that such breakpoints target specific genes leading to additional proliferative or survival advantage to the tumour cell. The present study illustrates the importance of characterization of such secondary rearrangements or breakpoints in order to understand the molecular basis of the acute phase in CML.

Given the progress in CML treatment due to the development of 1^st ^and 2^nd ^generation small molecules (TK-inhibitors) such as imatinib, nilotinib and dasatinib [15], targeting of such additional oncogenic events now represents a new challenge for future CML treatment. Even though currently the number of targeted therapies is still limited, it can be anticipated that in the future, combination therapies with imatinib and compounds targeted at the secondary changes can become a reality. Therefore, we propose a systematic testing of 3q26 aberrations in CML patients in blast crisis or CML patients with an evolutionary disease course. Since to date only a few target genes of the transcriptional repressor *EVI1 *are known [16], further research will be needed to identify more genes implicated in *EVI1 *pathogenesis thus opening the way for development of novel targeted therapeutics.

## Conclusion

In conclusion, we report on a CML patient in blast crisis presenting with a three-step variant Ph-positive chromosome rearrangement, involving the *EVI1 *locus. This report shows that the variant translocation can specifically target a second oncogene which most likely contributes to the more aggressive phenotype of the leukemia. Molecular analysis of t(9;22) variants is needed to understand its phenotypic consequences and to open the way to combined molecular therapies in order to counteract the secondary oncogenic effect which is unresponsive to imatinib treatment.

## Abbreviations

*ABL1*: ABeLson murine leukemia viral oncogene homolog 1; ALL: Acute Lymfoid Leukemia; AML: Acute Myeloid Leukemia; BC: Blast Crisis; BCR: Breakpoint Cluster Region; CML: Chronic Myeloid Leukemia; EAC: Europe Against Cancer; *EVI1*: Ecotropic Viral Integration site 1; FISH: Fluorescence In Situ Hybridisation; FITC: Fluorescein IsoThioCyanate; *MDS1*: MyeloDysplasia Syndrome 1; qRT-PCR: Quantitative Reverse Transcription Polymerase Chain Reaction; TK: Tyrosine Kinase; TRITC: TetramethylRhodamine IsoThioCyanate; WCP: Whole Chromosome Paint.

## Competing interests

The authors declare that they have no competing interests.

## Authors' contributions

ADW carried out the genetic studies and drafted the manuscript. AC is the patients referring physician. BC helped with *EVI1 *expression analysis, JD performed the blood cell counts, BV analyzed the *BCR-ABL1 *expressions and JP performed the immunophenotyping. BP, NVR and FS helped to draft the manuscript.

## Pre-publication history

The pre-publication history for this paper can be accessed here:


